# Inflammation induces neuro-lymphatic protein expression in multiple sclerosis brain neurovasculature

**DOI:** 10.1186/1742-2094-10-125

**Published:** 2013-10-14

**Authors:** Ganta Vijay Chaitanya, Seiichi Omura, Fumitaka Sato, Nicholas E Martinez, Alireza Minagar, Murali Ramanathan, Bianca Weinstock Guttman, Robert Zivadinov, Ikuo Tsunoda, Jonathan S Alexander

**Affiliations:** 1 Department of Molecular & Cellular Physiology, School of Medicine, Louisiana State University Health-Shreveport, 1501 Kings Highway, Shreveport, LA, 71130, USA; 2 Department of Microbiology and Immunology, School of Medicine, Louisiana State University Health-Shreveport, 1501 Kings Highway, Shreveport, LA, 71130, USA; 3 Department of Neurology, School of Medicine, Louisiana State University Health-Shreveport, 1501 Kings Highway, Shreveport, LA, 71130, USA; 4 Department of Neurology, State University of New York, Buffalo, NY, USA; 5 Department of Pharmaceutical Sciences, State University of New York, Buffalo, NY, USA; 6 Buffalo Neuroimaging Analysis Center, State University of New York, Buffalo, NY, USA

**Keywords:** Prox-1, Angiopoietin-2, VEGFR-3, VEGF-D, LYVE-1, Podoplanin/D2-40

## Abstract

**Background:**

Multiple sclerosis (MS) is associated with ectopic lymphoid follicle formation. Podoplanin^+^ (lymphatic marker) T helper17 (Th17) cells and B cell aggregates have been implicated in the formation of tertiary lymphoid organs (TLOs) in MS and experimental autoimmune encephalitis (EAE). Since podoplanin expressed by Th17 cells in MS brains is also expressed by lymphatic endothelium, we investigated whether the pathophysiology of MS involves inductions of lymphatic proteins in the inflamed neurovasculature.

**Methods:**

We assessed the protein levels of lymphatic vessel endothelial hyaluronan receptor and podoplanin, which are specific to the lymphatic system and prospero-homeobox protein-1, angiopoietin-2, vascular endothelial growth factor-D, vascular endothelial growth factor receptor-3, which are expressed by both lymphatic endothelium and neurons. Levels of these proteins were measured in postmortem brains and sera from MS patients, in the myelin proteolipid protein (PLP)-induced EAE and Theiler’s murine encephalomyelitis virus (TMEV) induced demyelinating disease (TMEV-IDD) mouse models and in cell culture models of inflamed neurovasculature.

**Results and conclusions:**

Intense staining for LYVE-1 was found in neurons of a subset of MS patients using immunohistochemical approaches. The lymphatic protein, podoplanin, was highly expressed in perivascular inflammatory lesions indicating signaling cross-talks between inflamed brain vasculature and lymphatic proteins in MS. The profiles of these proteins in MS patient sera discriminated between relapsing remitting MS from secondary progressive MS and normal patients. The *in vivo* findings were confirmed in the *in vitro* cell culture models of neuroinflammation.

## Background

Multiple sclerosis (MS) is an inflammatory demyelinating disease with neurodegeneration characterized by demyelinating plaques, neuronal, and axonal loss [1-3]. The precise etiology and pathophysiology for MS remain unknown. Many key steps in MS pathogenesis including the breakdown of the blood–brain barrier and the extravasation of immune cells into the brain parenchyma involve interactions with the vascular endothelium [4-12]. The enhanced spatial resolution of ultra-high field 7T magnetic resonance imaging (MRI) has demonstrated that >80% of white matter lesions in MS patients have a central vein (21) and natalizumab, an anti-α4 integrin monoclonal antibody that blocks cell-cell interactions between VLA-4 (α4β1 integrin, CD49d/CD29 heterodimer, also known as very late antigen-4) expressed on activated immune cells and vascular cell adhesion molecule (VCAM-1) expressed on the vascular endothelium is an approved drug for treating MS. Although the brain lacks 'classical’ lymphatics, interstitial fluid and solutes have been shown to drain along 150–200 nm wide 'lacunae’ in the basement membranes of arteries and capillary walls [13-18]. The role of any of lymphatic proteins in MS pathogenesis has not been extensively investigated.

Moreover, tertiary lymphoid organs (TLOs) with ectopic lymphoid follicles have been observed in the central nervous system (CNS) of MS patients as well as in animal models of MS [19-24]. The characteristic features of TLOs include compartmentalization of T and B cells, presence of lymphatic vessels, and high endothelial venules (HEVs) [24-30]. Th17 cells and B cells were shown to be the main contributors in the formation of these structures. However, the cellular mechanisms involved in their development and their function was not yet clear. It has been suggested that TLOs participate in antigen presentation and contribute to the progression of the disease to chronic stage (probably from RRMS to secondary-progressive MS (SPMS)) [24]. However, formation of HEVs has not been reported in MS [20,31,32] and it is not known whether these TLOs contain active lymphatic vascular structures. The lack of reliable lymphatic markers to distinguish between blood and lymphatic vasculature did not permit such studies until recently. The availability and validation of novel lymphatic marker proteins (podoplanin/D2-40, LYVE-1, VEGFR-3, VEGF-D, Ang-2) and transcription factors (Fox-C2, Prox-1) [33-36] have rapidly expanded our understanding of lymphatics and allow investigation of their roles in MS pathophysiology. While LYVE-1, podoplanin, and Fox-C2 are lymphatic-specific, Prox-1, VEGFR-3, VEGF-D, and Ang-2 also participate in neuronal function and development [37-42].

Our working hypothesis is that alterations in lymphatic proteins may play a critical role in tertiary lymphoid formation in MS. The purpose of this study was to investigate the role of lymphatic vessel endothelial hyaluronan receptor and podoplanin, which are specific to the lymphatic system, and prospero-homeobox protein-1, angiopoietin-2, vascular endothelial growth factor-D, and vascular endothelial growth factor receptor-3, which are expressed by both lymphatic endothelium and neurons in MS pathogenesis. Lymphatic protein expression was measured in postmortem brains and sera from MS patients, in the myelin proteolipid protein (PLP)-induced EAE and Theiler’s murine encephalomyelitis virus (TMEV) induced demyelinating disease (TMEV-IDD) mouse models and in cell culture models of neurovasculature inflammation.

## Methods

### Cell culture

Human brain endothelial cell line (HBMEC-3, provided by Dr. Anat Erdreich Epstein, Saban Research Institute at Children’s Hospital Los Angeles, University of Southern California, USA) was cultured in RPMI medium supplemented with 10% fetal calf serum (FCS) and 1% penicillin/streptomycin/amphotericin (PSA). Human neuronal like cell line (SHSY-5Y) was cultured in DMEM supplemented with 10% FCS and 1% PSA.

### Animal models of MS (PLP-EAE and TMEV-IDD) and EAE induction

All animal experiments were performed according to Institutional Animal Care and Use (ACUC) Committee guidelines at the Louisiana State University Health Sciences Center-Shreveport with approved animal protocols.

For PLP-EAE, three or four female SJL/J mice (4 weeks, Jackson Labs, Bar Harbor, ME, USA) were randomly chosen from a group of mice subcutaneously sensitized with 100 nmol of myelin proteolipid protein (PLP)^139–151^ peptide (VSLGKWLGHPDKF, United BioSystems Inc., Rockville, MD, USA) emulsified in complete Freund’s adjuvant, as described [43]. Briefly, both brains and sera from the randomly selected PLP-EAE mice were harvested on day 14 post sensitization. Clinical scores of PLP-EAE animals were evaluated daily based on the following scale: 0, no sign; 1, paralyzed tail; 2, mild hind limb paresis; 3, moderate hind limb paralysis; 4, complete hind limb paralysis; and 5, quadriplegia or moribund.

For TMEV-IDD, three or four mice were randomly chosen from a group of mice infected intra-cerebrally with 2×10^5^ plaque forming units (PFU) of TMEV. Brains and sera were harvested on day 35 after infection. Clinical scores of TMEV-IDD animals were evaluated daily based on examining impairment of righting reflex: proximal end of mouse tail was grasped and twisted to the right and then to left. The following scale was used: 0, a healthy mouse resists being turned over; 1, the mouse is flipped onto to its back but immediately rights itself; 1.5, the mouse is flipped onto its back but immediately rights itself on both sides; 2, the mouse rights itself in 1 to 5 s; 3, righting takes >5 s; 4, the mouse cannot right itself.

### MS specimens

De-identified postmortem MS brain specimens were obtained from the Human Brain and Spinal Fluid Resource Center (Neurology Research, Los Angeles, CA, USA). The banked control brain tissues included donors with brain disease. MS brain samples were obtained from patients clinically diagnosed with RRMS. Details of clinical diagnosis, gross, and micro-neuropathology of postmortem MS brain samples are indicated in Additional file 1: Table S1. De-identified samples of control, RRMS, and SPMS sera were obtained from the MS Center at the University at Buffalo.

### Western blots

Five microliters of PLP-EAE, TMEV-IDD, RRMS, SPMS, and respective controls’ serum specimens were immunoblotted for Prox-1, D2-40, LYVE-1, Ang-2, VEGFR-3, or VEGF-D. Control, PLP-EAE, TMEV-IDD mouse brains, and postmortem MS brain samples were homogenized in modified RIPA buffer (0.25 M sucrose, 50 mM Tris base, 150 mM NaCl, 1 mM EDTA, 1 mM MgCl_2_, 10 mM KCl, 1% NP40, and 1% Tween-20 supplemented with protease and phosphatase inhibitor cocktail-Sigma) and protein content was measured by the Lowry method. Equal amount of protein from these samples was separated on SDS-PAGE and immunoblotted with PROX-1, D2-40, LYVE-1, VEGFR-3, VEGF-D, and Ang-2.

### Antibodies

Angiopoietin-2 (Cat no. ab65835, Abcam), Podoplanin/D2-40 (Cat no. SIG-3730, Covance), LYVE-1 (for immunofluorescence) (Cat no. ab14917, Abcam), LYVE-1 (an in-house antibody), Prox-1 (Cat no. PRB-238C, Covance), VEGFR-3 (Cat no. AB1875, Chemicon), VEGF-D (Cat no. MAB286, R&D), Pan axonal neurofilament marker (Cat no. SMI311, Sternberger Monoclonals Inc.).

### Immunohistochemistry

Postmortem MS brain tissue was fixed in 4% paraformaldehyde and 3 μm sections were processed through alcohol/xylene followed by antigen retrieval solution (Pro-histo), washed, and incubated in primary antibodies against LYVE-1 and D2-40 for overnight at 4°C. Later sections were washed and incubated with super sensitive link label IHC detection system (Cat no. QP900-9L, Biogenex) for 30′ with three washes in between. Later sections were washed and developed using di-amino benzidine and photographed. All primary antibody dilutions were performed in pro-histo amplifying antibody dilution buffer (Pro-Histo, Cat no. AA3).

### Double immunofluorescence

Sections were washed and incubated in a cocktail of primaries against SMI311 and LYVE-1 overnight at 4°C. Negative controls included omitting primary antibodies. Later sections were washed three times and incubated in cocktail of AlexaFluor488 and Cy-3 conjugated secondary antibodies for 2 h at room temperature. All primary and secondary antibody dilutions were performed in pro-histo amplifying antibody dilution buffer. Later sections were washed and mounted using mounting media with DAPI and observed under Olympus fluorescent microscope.

### Cell ELISA

At confluence HBMEC3-brain endothelium and SHSY-5Y neurons were treated with 25 ng/mL of TNF-α, IL-1β, IFN-γ, and IL-3 for 24 h. After treatment, cell ELISA for podoplanin (D2-40), LYVE-1, PROX-1, VEGFR3, VEGF-D, and Ang-2 was performed according to previously published protocol [44]. Briefly, cells were fixed for 5 min with 1% PFA, washed, and incubated in primary antibodies diluted in HBSS/DPBS/5%FCS for 2 h at 37°C. Later cells were washed three times with HBSS/DPBS/1% FCS and incubated in HRP conjugated secondary antibodies for 1 h at 37°C. Later cells were washed three times and incubated in substrate combination of TMB/H_2_O_2_. Reaction was stopped by addition of 8N H_2_SO_4_ and the plates were read on an ELISA plate reader at 450 nm. Absorbance of untreated control cells was used as the baseline 100%. For Prox-1 cell ELISA, an additional step of treating cells with 0.25% Triton X-100 for 5′ was added after fixation to permeabilize the cells.

### Heat map and PCA

Principal component analysis (PCA) for expression data of marker proteins was conducted using R-software (v2.15.1, prcomp package). To study patterns of lymphatic biomarker expression in the brain, we converted densitometry data into heat maps, using 'R-software’ (R Development core team, 2012 [45]). We conducted 'supervised’ pair-wise comparisons of western blot data between control and MS samples [46] and drew heat map and dendrogram using R version 2.15.1 and the program package 'stats’ and 'pvclust’ (R Core team, 2012). We also conducted 'unsupervised’ PCA by entering western blot data of control and MS samples without grouping with R and the program package 'prcomp’ [47-49]. The proportions of variance and factor loadings for principal components (PC) were also calculated at the same time [47-49]. Heat maps and hierarchical clustering dendrograms were generated using the R-Software package.

### Statistical analysis

The independent samples *t* test with two-tailed *P* value was used to check significance between two groups, and one-way ANOVA with Dunnetts post-hoc test was used to assess the significance between more than two groups.

## Results

### Neuro-lymphatic proteins in postmortem RRMS brain and RRMS and SPMS sera

#### Expression of neuro-lymphatic proteins in postmortem MS brain

**Figure 1:**
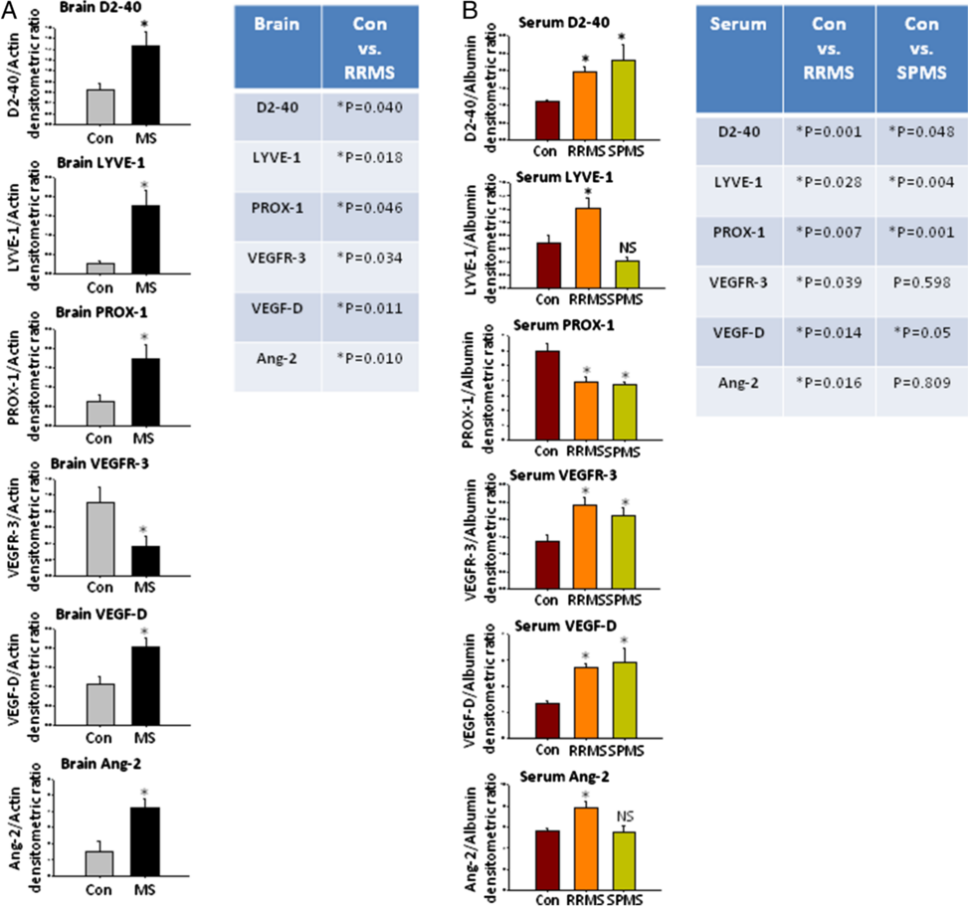
**Western blots of Neuro-Lymphatic proteins in MS brains and sera. (A)** Western blots from human control and MS postmortem brain tissue. Con=Postmortem human control brain tissue, MS=Postmortem human RRMS brain tissue. Con *n*=5, MS *n*=9, *P <0.05 considered significant. Table shows the *P* value between two groups. Two-way ANOVA with unpaired *T*-test between two individual groups. **(B)** Western blots of RRMS and SPMS patients’ serum. Table shows the *P* value between two groups. Con *n*=4, RRMS *n*=5, SPMS *n*=5. *P <0.05 is considered statistically significant. Two-way ANOVA with unpaired *T*-test between two individual groups.

Western blot analysis showed significant increase in the levels D2-40 (*P*=0.04), LYVE-1 (*P*=0.018), Prox-1 (*P*=0.046), VEGF-D (*P*=0.011), and Ang-2 (*P*=0.01) were observed in RRMS samples compared to controls. VEGFR-3 levels were significantly lower (*P*=0.034) in human MS brain tissue possibly indicating neuronal degeneration (Figure 1A, Additional file 2: Figure S1A).

#### Expression of neuro-lymphatic proteins in sera from RRMS and SPMS patients

We found increased expression of D2-40 (*P*=0.0018), LYVE-1 (*P*=0.028), VEGFR-3 (*P*=0.039), VEGF-D (*P*=0.014), and Ang-2 (*P*=0.0164) in RRMS sera, Prox-1 levels were decreased (*P*=0.0075) in RRMS serum *versus* controls (Figure 1B, Additional file 2: Figure S1B).

D2-40 and VEGF-D (*P*=0.05) were increased in SPMS sera (*P*=0.048) whereas LYVE-1 (*P*=0.004) and Prox-1 (*P*=0.001) decreased. No differences were found in Ang-2 or VEGFR-3 levels in SPMS sera (Figure 1B, Additional file 2: Figure S1B).

### Immunohistochemistry of lymphatic specific proteins LYVE-1 and D2-40 in RRMS brains

Since we observed a significant increase in lymphatic specific D2-40 and LYVE-1 expression in western blot analysis, we performed immunohistochemistry for D2-40 and LYVE-1 to understand the tissue distribution of these proteins.

**Figure 2:**
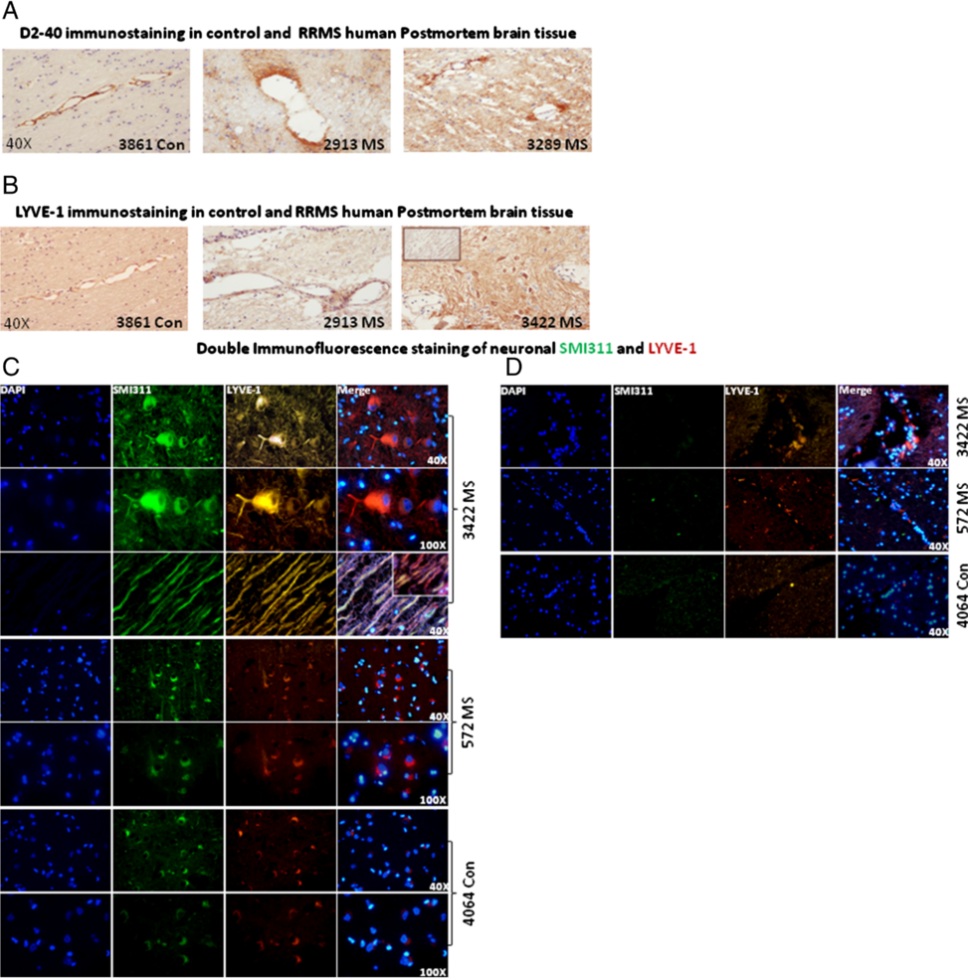
**Immunostaining of LYVE-1 and Podoplanin (D2-40) in MS brains. (A)** D2-40 Immunostaining of control and MS human postmortem brain tissue. D2-40 immunostaining was mainly localized to the inner wall of brain endothelium in normal brain tissue sections and at perivascular inflammatory regions in MS brain tissue sections. Magnification 40×. **(B)** LYVE-1 immunostaining in control and MS human postmortem brain tissue. LYVE-1 immunostaining was not confined to vasculature in the MS tissue since, neuronal like cells were found to be LYVE-1^+^ in sample numbers 572, 3422, and 3816. Inset shows the axonal positivity of LYVE-1 in MS brain tissue. Magnification 40×. **(C)** Double immunofluorescence analysis of LYVE-1 and SMI311 in patient samples 3422 and 572. We observed a strong co-localization of SMI311 (recognizes both neuronal bodies and axons) stained in green (Alex-488) with LYVE-1 stained in red (cy3) in RRMS brain tissue samples. Furthermore, SMI311 was also observed to be co-localized on axons. LYVE-1 staining was primarily confined around the axons indicating oligodendrocyte/myelin positivity. Inset shows a higher magnification of SMI311 co-localization with axonal structures in MS 572 (magnification 100×). **(D)** LYVE-1 immunofluorescence also showed higher numbers of LYVE-1^+^ immune cell infiltrates in the MS brain vasculature (572, 3422) compared to controls (4064).

Immunostaining for D2-40 and LYVE-1 in RRMS brain tissue showed prominent vascular staining. MS plaques showed greater D2-40 immunostaining compared with non-plaque regions or control brain tissue (Figure 2A, Additional file 3: Figure S2A). While normal brains tissue showed D2-40 immunostaining closely associated with the vascular lining of blood vessels, MS brain tissue was intense with perivascular parenchyma. Immunohistochemical staining of MS brain sections showed increased D2-40 staining intensity on and around brain microvessels consistent with D2-40 as a possible marker of microvessel inflammation in MS brain tissue.

LYVE-1 vascular immunostaining was also more intense in MS than in controls which showed low LYVE-1 immunoreactivity. Apart from vessel structures, neuronal LYVE-1 staining was observed in several MS samples (3816, 3422, 572, and 2946). Brain parenchyma in MS plaques also stained intensely for LYVE-1 (Figure 2B, Additional file 3: Figure S2B).

**Figure 3:**
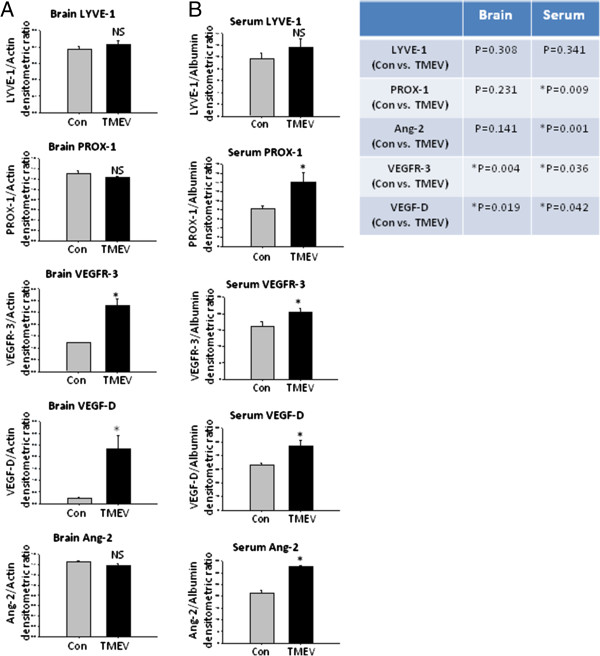
**Western blots of Neuro-lymphatic proteins in TMEV-IDD mice brains and sera. (A)** Western blots of PLP-EAE brains. Con=control, CFA=Complete Freund’s Adjuvant treated mice. CFA+PLP=Complete Freund’s Adjuvant + Myelin proteolipid protein treated mice. Con *n*=3, CFA *n*=3, CFA+PLP *n*=4. Table shows the *P* value between two groups. *P <0.05 is considered statistically significant. Two-way ANOVA with unpaired *T*-test between two individual groups. **(B)** Western blots PLP-EAE sera. Con *n*=3, CFA *n*=3, CFA+PLP *n*=4. Table shows the *P* value between two groups. *P <0.05 is considered statistically significant. Two-way ANOVA with unpaired *T*-test between two individual groups.

Apparent neuronal LYVE-1 staining was found in MS patient samples 572 and 3422, and was confirmed by double labeling with the neuronal marker 'SMI311’ (Figure 2C). Further, distinctive peri-axonal LYVE-1 staining showing myelin positivity was observed (sample 3422). Figure 3B (MS sample 3422) inset shows LYVE-1 immunohistochemistry in a consecutive section which confirms axonal identity. Double-immunofluorescence confirmed that LYVE-1 staining was co-localized with peri-axonal oligodendrocyte/myelin (Figure 2C). We also observed higher numbers of LYVE-1^+^ immune cells in the vasculature in MS brain tissue compared to controls (Figure 2D).

### Neuro-lymphatic protein alterations in TMEV-IDD

**Figure 4:**
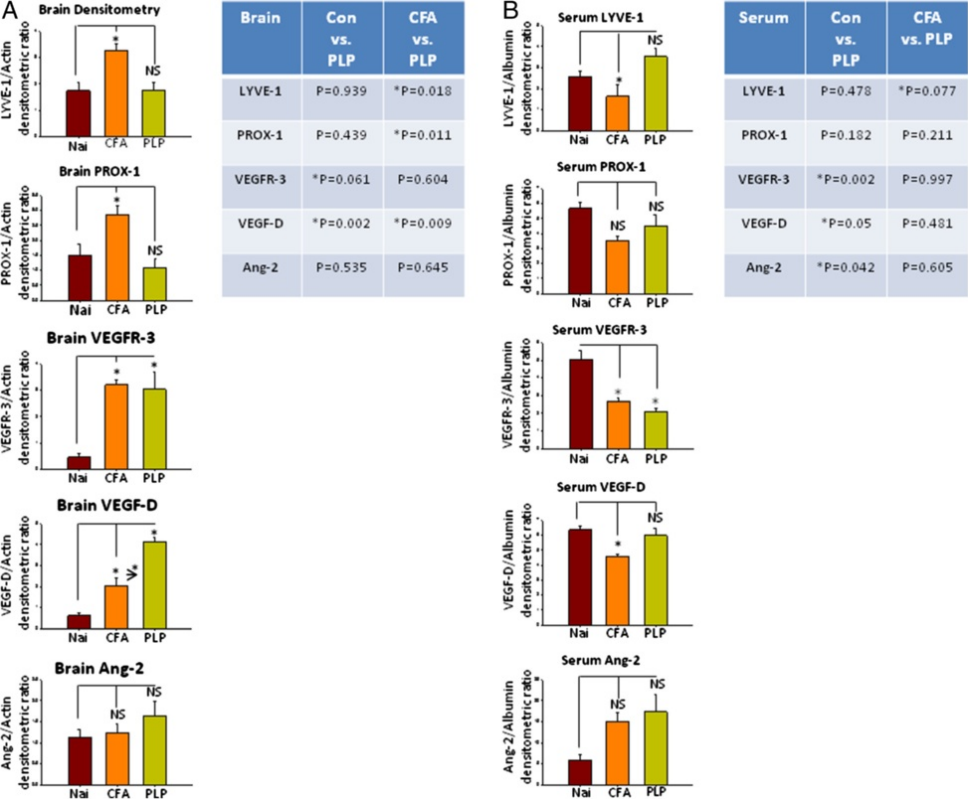
**Western blots of Neuro-Lymphatic proteins in PLP-EAE mice brains and sera. (A)** Western blots from TMEV-IDD brains. Nai=Naïve, TMEV=TMEV-IDD. Naïve *n*=3, TMEV *n*=3. Table shows the *P* value between two groups. *P <0.05 is considered statistically significant. Two-way ANOVA with unpaired *T*-test. **(B)** Western blots TMEV-IDD sera. Naïve *n*=3, TMEV *n*=3. Table shows the *P* value between two groups. *P <0.05 is considered statistically significant. Two-way ANOVA with unpaired *T*-test.

TMEV-IDD mouse brains had significantly higher levels of VEGFR3 (*P*=0.004) and VEGF-D (*P*=0.019*)* compared to controls*.* No difference in Prox-1, LYVE-1, or Ang-2 levels were observed in brains of TMEV-IDD compared with controls (Figure 4A, Additional file 4: Figure S3A).

We observed a significant increase in the levels of Prox-1 (*P*=0.009), VEGFR-3 (*P*=0.036), VEGF-D (*P*=0.042), and Ang-2 (*P*=0.001) in the sera of TMEV-IDD mice. No difference in levels of LYVE-1 was observed (Figure 4B, Additional file 4: Figure S3B).

### Neuro-lymphatic protein alterations in PLP EAE

An interesting pattern of 'lymphatic’ protein expression was observed in the brains of naïve, CFA, and CFA+PLP-EAE mice.

VEGF-D was the only protein (naïve *vs.* CFA *vs.* CFA+PLP) that showed significant increase between naïve, CFA, and CFA+PLP-EAE brains (naïve *vs.* CFA, *P*=0.024; CFA *vs.* CFA+PLP, *P*=0.009; naïve *vs.* CFA+PLP, *P*=0.002). No difference in Ang-2 levels was observed between any of groups (Figure 3A, Additional file 5: Figure S4A).

CFA induced significantly higher levels of LYVE-1 (*P*=0.013) and Prox-1 (*P*=0.045). No significant differences in LYVE-1 or Prox-1 expression were observed in the brains of naïve and CFA+PLP-EAE mice.

CFA and CFA+PLP-EAE mice brains had significantly higher levels of VEGFR3 (naïve *vs.* CFA, *P*=0.003; naïve *vs*. CFA+PLP-EAE, *P*=0.019) compared to naïve controls. However, no difference between CFA *versus* CFA+PLP-EAE in VEGFR3 levels was observed.

We also observed changes in 'lymphatic’ protein expression in the sera of naïve, CFA, and CFA+PLP-EAE mice. While CFA significantly decreased levels of LYVE-1 (*P*=0.046) and VEGF-D (*P*=0.003), no difference was observed in LYVE-1 and VEGF-D levels between naïve and CFA+PLP-EAE mice. Both CFA and CFA+PLP-EAE mice sera had lower levels of VEGFR3 (naïve *vs.* CFA, *P*=0.010; naïve *vs.* CFA+PLP, *P*=0.002) compared to controls. However, no difference between CFA *versus* CFA+PLP-EAE in VEGFR3 levels was observed. No difference in Prox-1 or Ang-2 levels was observed between any of the groups (Figure 3B, Additional file 5: Figure S4B).

Comparisons of these serum and brain tissue lymphatic proteins in mouse models and human MS are shown in Table 1.

**Table 1:** Western data of D2-40, LYVE-1, Prox-1, VEGFR-3, VEGF-D and Ang-2 levels in MS and its animal models

	**D2-40**	**LYVE-1**	**PROX-1**	**VEGFR-3**	**VEGF-D**	**Ang-2**
MS brain (RRMS)	↑	↑	↑	↓	↑	↑
TMEV brain	NA	-	-	↑	↑	-
PLP brain^a^	NA	-	-	-	↑	-
RRMS serum	↑	↑	↓	↑	↑	↑
SPMS serum	↑	-	↓	↑	↑	-
TMEV serum	NA	-	↑	↑	↑	↑
PLP serum^a^	NA	-	-	-	-	-

Con vs. RRMS human brain samples; Con vs. SPMS and RRMS serum samples; Control vs. TMEV-IDD; and naïve vs. CFA, PLP serum and brain samples. For naïve vs. CFA vs. CFA+PLP-EAE only proteins that showed statistical difference between all the 3 groups were shown.

^a^CFA + PLP is significantly different from both Naïve and CFA alone.

### PCA analysis

**Figure 5:**
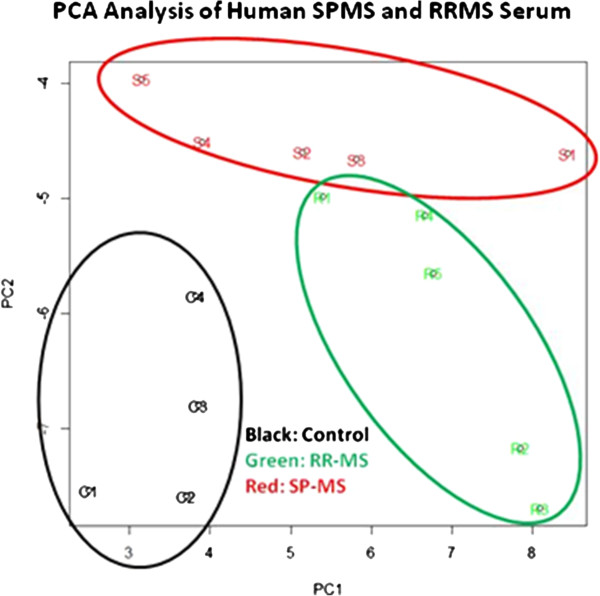
PCA ordination diagram of the densitometric values from western analysis of six lymphatic proteins in the sera of con, RRMS, and SPMS patients clustered the samples distinctly separate from each other.

We hypothesized that lymphatic markers might represent important biomarkers for demyelinating diseases. We next conducted PCA of lymphatic markers to determine whether controls, RRMS, or SPMS samples could be separated into three distinct groups: Con, RRMS, and SPMS. PCA was applied to immunoblot densitometry data of six lymphatic markers in control, RRMS, and SPMS sera. PC1 and PC2 accounted for >90% of variance (PC1, 61%; PC2, 28%). RRMS and controls could be separated along the axis defined by PC1, but not PC2, indicating that PC1 can explain an important part of the variance that separates RRMS from controls. RRMS and SPMS could be separated along the axis defined by PC2 but not PC1. Thus, serum lymphatic markers, as a whole, can be used to separate these three groups (Figure 5).

To determine which serum lymphatic markers contribute to PC1 and PC2, we next looked at factor loading (Additional file 6: Figure S5). We found that Prox-1 was negatively associated with both PC1 and PC2 values. This is consistent with western blot analysis, in which Prox-1 expression was decreased in both RRMS and SPMS. Interestingly, Ang-2 and LYVE-1 were positively associated with PC1 values and negatively associated with PC2 values. Again, these results were consistent with the findings from the inter-group statistical comparisons, in which Ang-2 and LYVE-1 levels were increased in RRMS but not SPMS.

### Inflammatory cytokines induce differential neuro-lymphatic protein expression on neurovasculature *in vitro*

In order to understand the role of inflammation in the induction of these common neuronal/lymphatic proteins in neurovasculature, human neurons, and brain endothelial cells were treated with inflammatory cytokines including TNF-α, IL-1β, and IFN-γ for 24 h. IL-3 was used as a positive treatment condition based on previously published data [50].

#### HBMEC-3 human brain endothelium

**Figure 6:**
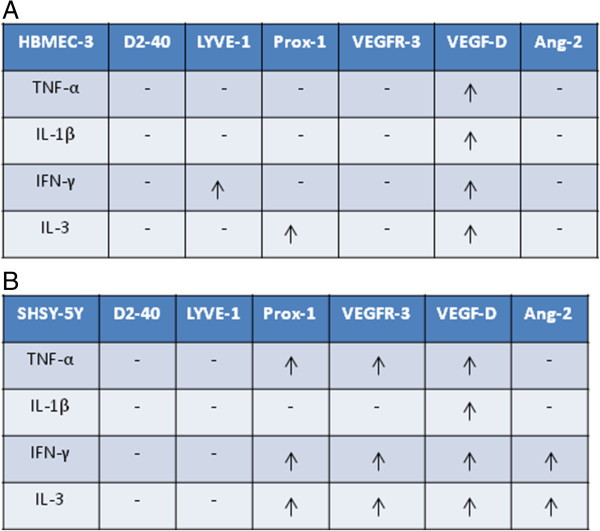
**Cell ELISA of cytokine induced Neuro-Lymphatic protein expression on human brain neurovasculature *in vitro*. (A)** Cell ELISA of neuronal/lymphatic proteins in HBMEC-3 brain endothelial cells. IFN-γ= Interferon-gamma, IL-1β= Interleukin-beta, TNF-α= Tumor necrosis factor-alpha. *P <0.05, #*P* close to significance (*n*=6-8). **(B)** Cell ELISA of neuronal/lymphatic proteins in SHSY-5Y neurons. IFN-γ= Interferon-gamma, IL-1β= Interleukin-beta, TNF-α= Tumor necrosis factor-alpha. *P <0.05, #*P* close to significance (*n*=6-8).

All the treatment conditions including TNF-α (*P*=0.042), IL-1β (*P*=0.008), IFN-γ (*P*=0.001), and IL-3 (*P*=0.001) induced VEGF-D expression in HBMEC-3. IFN-γ induced significant increase in LYVE-1 expression (*P*=0.001), whereas IL-3 induced significant increase in Prox-1 expression (*P*=0.007). None of the treatment condition induced D2-40, Ang-2, or VEGFR-3 expression in human brain endothelium (Figure 6A, Additional file 7: Figure S6A).

#### SHSY-5Y human neurons

Similar to HBMEC-3 all the cytokines including TNF-α (*P*=0.0001), IL-1β (*P*=0.002), IFN-γ (*P*=0.002), and IL-3 (*P*=0.001) induced higher levels of VEGF-D. In addition to IL-3 (*P*=0.002), TNF- α (*P*=0.001), and IFN-γ (*P*=0.03) induced higher levels of Prox-1 in SHSY-5Y neurons. VEGFR-3 expression in SHSY-5Y neurons was induced by TNF-α (*P*=0.01), IFN-γ (*P*=0.03), and IL-3 (*P*=0.002). IFN-γ (*P*=0.003) and IL-3 (*P*=0.01) induced significantly higher levels of SHSY-5Y Ang-2 expression (Figure 6B, Additional file 7: Figure S6B). None of the treatment conditions induced D2-40 or LYVE-1 expression.

## Discussion

While the functional role of neuronal LYVE-1 is still not clear, several of the common neuro-lymphatic proteins in the study including Prox-1, VEGFR-3, Ang-2, and VEGF-D are indispensable for brain development and neuronal function. For example, induction of neuronal specific Prox-1 and Mash-1 are crucial for the differentiation of neural stem cells in the developing brain [42]. Prox-1 also controls Notch-1 dependent neurogenesis in spinal cord [51]. In the developing rat brain VEGFR-3 expression has been observed in forebrain, retina, and cerebellum [52,53]. Interestingly its expression is enhanced in the subventricular zone of the stroke lesions in rat brain [54]. The expression of VEGFR-3 in subventricular zone suggests its participation in stem cell signaling. Even though, both VEGF-C and VEGF-D are ligands for VEGFR-3, VEGF-C inhibition severely reduces the proliferation of VEGFR-3 expressing neural progenitor cells in both xenopus tadpoles and in mice [55], indicating that VEGF-C: VEGFR-3 axis functions in the stem cell signaling in developing brain as well as in the pathophysiology of CNS diseases like stroke. Since, VEGF-C and VEGFR-3 signaling has been shown to modulate innate and adaptive immune responses in experimental obliterative bronchiolitis [56], we expect that this VEGF-C: VEGFR-3 axis might also play a key role in the pathophysiology of MS.

VEGF-D an important ligand for VEGFR-3 signaling also plays an important role in the differentiation of human pluripotent stem cells to dopaminergic neurons [57]. Furthermore, VEGF-D plays an important role in neuronal synaptic activity, dendritic length, and in the maintenance of complex dendrite arborization via calcium dependent calcium-calmodulin-dependent protein kinase IV signaling [40]. Inhibition of mouse hippocampal VEGF-D result in memory deficits indicating that calcium dependent VEGF-D signaling is highly important for neuronal function and cognition [40]. Apart from its role in vasculature Ang-2 was also shown to be involved in the regulation of cortical neurogenesis in the telencephelon [39]. Inhibition of Ang-2 in neuronal progenitor cells effects neuronal migration, intermediate progenitor cells, and the surrounding vasculature in embryonic brain [39]. Furthermore, Ang-2:Tie2: matrix metalloproteinases interactions play an important role in the differentiation of neural progenitor cells to neuronal lineage and migration of neuronal progenitor cells in subventricular zone after stroke [38].

Our current findings showing that neuroinflammatory diseases like MS can induce neuro-lymphatic protein expression in the CNS gain more importance based on the recent report showing a functional glymphatic system involved in the clearance of fluids from the CNS diseases [58-60]. While astrocytic aquaporin-4 plays a functional role in the transparenchymal clearance of interstitial solutes from the brain [60], it still remains unclear whether astrocytes also express these common 'neuro-lymphatic’ proteins in inflamed CNS. More importantly, whether this common neuro-lymphatic protein expression is casual bystander in the CNS inflammation or it participates in interstitial solute clearance function of glympahtic system needs further investigation. While the functional role of these neuro-lymphatic protein expressions in pathophysiology of CNS inflammation needs to be investigated, physiologically these proteins play crucial role in the CNS development and neuronal function.

Chronic autoimmune diseases like rheumatoid arthritis, autoimmune thyroiditis, myasthenia gravis, Sjogren’s syndrome, and MS are characterized by the formation of ectopic follicle like structures and TLO structures in the affected organs [19,20,23,24,30-32,61-67]. These TLOs sustain inflammation via pro-inflammatory mediators, participate in antigen presentation, generate auto-antibodies and autoreactive T and B cells, and aggravate the disease pathology. One of the main features of these TLOs is the presence of lymphatic vessels and expression of lymphoid chemokines including CCL12, CCL19, CCL21, and CXCL13 which are shown to participate in lymphangiogenesis and lymphatic and immune interactions [24,31,32,61,68-70]. It has been suggested that the number of TLOs correlate with the disease pathology [23,24]. Even though presence of lymphatics is one of the characteristic features of TLOs, it is still not clear whether lymphatic vasculature is present in the TLOs of MS brain/spinal tissue [31,32,71]. However, we expect that a correlation can be drawn between the neuronal/lymphatic protein alterations (in the brain/spinal cord tissue) with TLOs formation, disease severity, and type of MS.

One of the most important results of our study was the observation that neurons in MS some patients express LYVE-1. While the role of LYVE-1 in lymphatic vessels still awaits investigation, neuronal LYVE-1 expression suggests its potential physiological and pathological importance in MS. Whether neuronal LYVE-1 is expressed in CNS diseases besides MS also needs investigation. More importantly, we observed dramatically higher numbers of LYVE-1^+^ immune cells infiltrates in the MS brain vasculature compared to normal brain vasculature. Previous reports have shown CD11b^+^ myeloid cells express LYVE-1 and Prox-1 only when attached to the tumor vasculature but not unattached, indicating that vascular-immune interactions might play a prominent role in the expression of these proteins in the immune cells [72-74]. This might result in more complex interactions between infiltrating immune cells and MS neurovasculature in lymphoid TLO formation in MS. In addition to LYVE-1 immunostaining, we observed a distinct D2-40 expression by immunohistochemistry on normal brain blood vessels and an intense D2-40 staining on and around microvessels (perivascular positivity) in MS brains suggesting the use of D2-40 as an inflammatory endothelial marker. Since podoplanin^+^ Th17 cells are shown to be the main contributors of TLOs formation [20,23,24], we expect that in MS an increased D2-40 staining around the vasculature may represent future TLOs formation site due to the possible accumulation of Th17 cells at the sites of vascular entry. While TLOs formation was noticed in the brains of SPMS patients but not in primary-progressive MS (PPMS) or RRMS by Serafini et al. [19], intracerebral expression of CXCL13 and BAFF was shown to be accompanied by formation of lymphoid like structures in animal model of relapsing EAE [21].

However, since RRMS was described to have no TLOs formation and we observe significant increase in these protein profiles in both serum and brains of RRMS, we wanted to see if neurovasculature in MS might participate in the induction of these proteins under inflammatory environment. Since we observed an increase in brain Prox-1 levels and IL-3 can re-induce expression of Prox-1 and podoplanin in blood endothelial cells [50], we expect that increased IL-3 levels in MS pathology might provoke these changes and deserves further investigation. This was based on the recent study that shows that IL-3 can induce lymphatic specific Prox-1 in blood endothelium [50]. Taken this into consideration, we hypothesized that increased inflammatory environment in the MS brains might induce alterations in these proteins in neurovasculature. To confirm our hypothesis we performed cell ELISA using neuroinflammatory cytokines including IL-3. We observed a differential expression profile of these proteins in human neurons and human brain endothelium suggesting complex interactions of pro-inflammatory mediators with neurovasculature can induce alterations in these neuronal/lymphatic proteins in MS brain neurovasculature.

Even though, we observed distinct brain endothelial D2-40 staining in control and MS brains, none of the *in vitro* stimuli showed an induction in D2-40 expression indicating more complex roles of cytokine/chemokine/growth factor signaling networks and infiltrating immune cells in MS CNS. Of all the proteins under study, VEGF-D showed significant induction in MS and MS animal models (except PLP-EAE serum) and in neurovascular inflammatory conditions *in vitro*. It was well known that VEGF-D is one of the most potent lymphangiogenic factors [74]. Based on the result VEGF-D is the only growth factor that is altered in all these conditions, we expect a much complex role of VEGF-D in MS pathophysiology. We expect that VEGF-D might also participate in formation and sustaining of TLOs in MS disease. Further experiments to correlate VEGF-D levels with TLOs formation and gain/loss of VEGF-D function studies in MS will shed more light on its role in modulating neurovascular damage in the pathophysiology of MS. Furthermore, since some of these lymphatic specific proteins are expressed by neurons and blood vessels, the functions of these proteins in MS might be divergent, affecting the functions of neurovascular interactions in the brain and CNS-immune interactions. While our study showed several important vascular (blood and lymphatic) and common neuronal and lymphatic marker aberrations in the MS and its animal models, the functional roles of these proteins in the etiology, development, and progression of MS remains an important area of investigation.

Results from our western blotting analysis showed that the expression levels of common neuronal/lymphatic proteins in sera were significantly altered in demyelinating diseases: MS, PLP-EAE, and TMEV-IDD. Using the densitometry data from immunoblotting experiments, we conducted unsupervised PCA to determine whether controls and disease samples could be separated into different groups by expression patterns of sets of these neuronal/lymphatic proteins ('unsupervised’ analysis, conducted without information on samples; grouping was conducted post-analysis). Since PCA of these proteins resulted in grouping between controls and demyelinating diseases (for example, PCA of sera among control, RRMS, and SPMS clearly separated into three groups), these proteins may be used as a 'marker panel’ to discriminate between controls and disease samples. While our current results require further validation using larger sample size, our results suggest that a set of these 'protein panel’ in serum represent a novel, powerful panel which would discriminate between healthy subjects, RRMS, SPMS, and possibly PPMS without sampling cerebrospinal fluid or brain tissue. Correlating data from both mice and humans, PCA can help us to find effective markers which detect MS and provide an etiological basis of disease. Besides the potential advantage of PCA to identify proteins common to these pathologies that could serve to differentiate MS subtypes (with much larger sample size and measurement of the accurate protein levels) the functional roles of these proteins in MS pathophysiology deserves further investigation.

## Conclusions

Our study shows that RRMS pathophysiology is also accompanied with alterations in common neuronal/lymphatic protein expression and that inflammation induces the expression of these common neuronal/lymphatic proteins significantly higher in neurons and in brain endothelium. PCA results on the serum samples from RRMS and SPMS indicated that the expression of neuro-lymphatic protein profiles can separate both these MS types from normal patients. However, we were limited by small sample size and lack of SPMS brain samples. Larger studies that include PPMS samples are needed to assess whether these proteins can be used as a panel for differentiating MS subtypes.

## Competing interests

The authors declare that they have no competing interests.

## Authors’ contributions

GVC designed and performed experiments, analyzed and interpreted the data, wrote and edited the manuscript. SO performed animal experiments and bioinformatics analysis. FS and NEM performed animal experiments. AM, MR, BWG, and RZ provided MS serum samples and edited the manuscript. IT and JSA designed and edited the manuscript. All authors read and approved the final manuscript.

## Supplementary Material

Additional file 1: Table S1 Details of clinical diagnosis, gross, and micro-neuropathological information and other neuropathological symptoms of the postmortem MS patients’ brain samples. Click here for file

Additional file 2: Figure S1 **(A)** Western blot images of D2-40, LYVE-1, Prox-1, VEGFR-3, VEGF-D, and Ang-2 in control and RRMS brain tissue samples. Con *n*=5, RRMS *n*=9. **(B)** Western blot images of D2-40, LYVE-1, Prox-1, VEGFR-3, VEGF-D, and Ang-2 in control, RRMS, and SPMS serum samples. Click here for file

Additional file 3: Figure S2 **(A)** Additional D2-40 immunostained pictures of human postmortem control brain samples (3912, 4064, 4135, and 3805) and MS brain samples (3816, 3840, 572, and 3422). D2-40 immunostaining is intense and focused at the perivascular endothelial inflammatory region and parenchyma in all MS samples. **(B)** Additional LYVE-1 immunostained pictures of human postmortem control samples (3912, 4064, 4135, and 3805) and MS brain samples (3816, 3840, 572, and 2946). Brain parenchyma stained positive for intensively positive for LYVE-1 in MS sample 2946. Magnification 40×. Click here for file

Additional file 4: Figure S3 **(A)** Western blot images of LYVE-1, Prox-1, VEGFR-3, VEGF-D, and Ang-2 in randomly chosen control and TMEV-IDD mice brain tissue samples. Con *n*=3, TMEV-IDD *n*=3. **(B)** Western blot images of LYVE-1, Prox-1, VEGFR-3, VEGF-D, and Ang-2 in corresponding serum samples from control and TMEV-IDD mice. Click here for file

Additional file 5: Figure S4 **(A)**Western blot images of LYVE-1, Prox-1, VEGFR-3, VEGF-D, and Ang-2 in randomly chosen naïve, CFA-treated, and CFA+PLP-EAE mice brain tissue samples. Naïve *n*=3, CFA *n*=3, CFA+PLP-EAE *n*=4. **(B)** Western blot images of LYVE-1, Prox-1, VEGFR-3, VEGF-D, and Ang-2 in corresponding serum samples from naïve, CFA, and CFA+PLP-EAE mice. Click here for file

Additional file 6: Figure S5 Plot of the eigenvalues that reflect the variance of the principal components showed that >90% of the variance in this matrix of lymphatic proteins is contained in the first two principal components (PC1, 61%; PC2, 28%). The factor loading on the eigenvalues for PC1 and PC2 reflect the amount of variance shared by the parameter with the PC1 and PC2 values. Click here for file

Additional file 7: Figure S6 **(A)** Bar graphs showing the cytokine (TNF-α, IL-1β, IFN-γ, and Il-3) mediated neuro-lymphatic protein expression profiles in HBEMC-3 brain endothelial cells. **(B)** Bar graphs showing the cytokine (TNF-α, IL-1β, IFN-γ, and Il-3) mediated neuro-lymphatic protein expression profiles in SYSY-5Y neurons. Click here for file
