# Supplementary material for: Inflammation induces neuro-lymphatic protein expression in multiple sclerosis brain neurovasculature
Source: J Neuroinflammation. 2013 Oct 14;10:125. doi: 10.1186/1742-2094-10-125 (PMC3854084; doi:10.1186/1742-2094-10-125)
Supplement: Additional file 1: Table S1 — Details of clinical diagnosis, gross, and micro-neuropathological information and other neuropathological symptoms of the postmortem MS patients’ brain samples. [file 1742-2094-10-125-S1.pdf]

HSB Sample no	Age	Sex	Structure	Neuropathology Macroscopic description	Neuropathology Microscopic description	Sections	Clinical diagnosis	Clinical cause of death
2946	59	M	Occipital Plaque	Well developed central grey nuclei, ventricles are of appropriate size and lined by smooth thin ependyma. Meninges, vascular structures, ependyma and cranial nerves show no abnormalities. Substantia nigra and locus ceruleus are well pigmented, cerebellar sections have anatomically arranged grey and white matter with a dark tan. 0.5x0.3 cm discoloration of cerebellar white matter.	Parietal lobe demyelination, axonal loss in periventricular white matter and sparse, focal chronic perivascular inflammation	Peri-ventricular white matter, Parietal lobe	PPMS, Dementia	Pneumonia due to MS
3185	50	M	Occipital Plaque	Bilateral, very irregular periventricular plaques. Similar areas of plaques in corpus callosum, left internal capsule, temporal lobe white matter, pons, and cerebellar white matter. No apparent softening, discoloration, hemorrhage, mass or other lesion. Junction between cortex and white matter is well demarcated. Lateral cerebral ventricles are normal in size and shape. Normal striatum, lentiform nucleus, hippocampus and thalamus. No atrophy of the cerebellar folia. No atherosclerosis.	Demyelinating plaque, extensive decrease in intra-plaque oligodendrocyte cellularity, nearly complete demyelination, no associated perivascular cuffing and the adjacent cortex and white matter is unremarkable. Chronic MS plaque formation	Peri-ventricular white matter and adjacent cortex	PPMS, Hypothoroidism	Respiratory failure due to aspiration pneumonia, advanced MS
3509	74	F	Frontal plaque	No softening, discoloration, hemorrhage, mass or other lesion. No cortical atrpphy. Atrophic hippocampus, normal white matter. Well demarcated junction between cortex and white matter. Normal striatum, lentiform nucleus, amygdala and thalamus. No atrophy of cerebellar folia. A horizontal plaque in the right inferior pontine tegmentum in the brain stem. Basilar cerebral vasculature shows mild atherosclerosis.	Small focus of complete demyelination. Mild oligodendrocyte and relatively little axonal loss. Significant gliosis, macrophage activity or lymphocyte activity is not present.	Ventral pontine tegmentum/ dorsal basilar pons	PPMS, Hypertension	Cardiac arrest
3816	47	F	Occipital Plaque	Extensive irregular demyelinating periventricular plaque formation throughout the extent of the body of the right and left lateral ventricles with satellite extension into the respective corona radiate and the left basal ganglia. Similar changes in brainstem tegmentum. No apparent softening, discoloration, hemorrhage, mass or other lesion. No atrophy of cerebellar folia or significant atherosclerosis.	Plaque formation with extensive axonal loss and extensive demyelination, Near complete decrease in oligodendrocyte density within plaque and light increase in density along the periphery, mild to moderate gliosis, no prominent macrophage activity and perivascular lymphocytic cuffing.	Perivascular white matter	PPMS, Deep brain stimulator, Neurogenic baldder, tremor in head and neck	Respiratory failure without active infection, end stage MS
3840	61	F	Frontal plaque	Small patchy areas of demyelination in the middle cerebellar peduncle and in midbrain peri aqueductal area. No apparent softening discoloration, hemorrhage, mass or other lesion. Normal striatum, lentiform nucleus, and thalamus. No atrophy of the cerebellar folia. No significant atherosclerosis	Early plaque formation with mild axonal loss and minimally decreased oligodendrocyte density, moderate demyelination, no significant gliosis, increased macrophage activity and perivascular lymphocyte cuffing	Left middle cerebellar peduncle	PPMS, Optic neuritis, Chronic urinary tract infection, Depression, deep vein thrombosis, decubitus ulcer, GERD, Pneumonia.	Respiratory failure due to advanced MS
572	65	F	Occipital Plaque	---	Numerous foci of demyelination in the optic nerves, cerebral white matter and spinal cord.		RR MS	-

2931	73	M	Parietal plaque	Subcortical and deep white matter is remarkable for grey periventricular discolorations in the frontal and parietal lobes. Well developed central grey nuclei. Ventricles are of appropriate size and lined by a smooth thin ependyma. Meninges, vascular structures, ependyma and cranial nerves are not well represented but show no abnormalities. Substantia nigra and locus ceruleus are well pigmented. The cerebellar section has anatomically arranged grey and white matter structures and shows no additional abnormalities.	Demyelination and axonal loss with mild chronic perivascular inflammation	Frontal and periventricular white matter	RRMS	-
3289	54	F	Occipital Plaque	Extensive irregular bilateral periventricular plaques involving both lateral ventricles with the most intense involvement in the parietal and occipital lobes with decreasing involvement anteriorly. There is also an isolated left subcortical plaque. The cortical mantel is normal and good demarcation between the gray and white matter. The remaining areas are remarkable. No atherosclerosis.	Demyelinating plaque formation with demyelination and axonal loss, near complete loss of oligodendrocytes, presence of scattered astrocytes in the plaque with very reactive gliosis at the plaque margin, presence of numerous perivascular hemosiderin laden macrophages,adjacent white matter shows a slight increase in cellularity with mild perivascular inflammation.	Periventricular plaque	RRMS	Acute cardiac arrest
3413	38	F	Occipital Plaque	No apparent softening, discoloration, hemmorrage, mass or other lesion. Junction between the cortex and white matter is well demarcated. Large irregular periventricular plaques bilaterally extending from the frontal to the occipital poles. Some areas extend into the subcortical white matter. Irregular plaque formation in the caudal midbrain tegmentum. Normal straitum, lentiform nucleus, hippocampus, and thalamus normal. No atrophy of the cerebellar folia. The basilar vasculature shows minimal atherosclerosis.	Perivascular demyelination characterized by decreased axonal density and complete demyelination, mild to severe gliosis and oligodendrocyte loss, no associated macrophage activity or perivascular lymphocytic cuffing, adjacent white matter and cortex are unremarkable.	Perivascular white matter	RRMS	unexpected death by suffocation
3422	62	M	Cerebellar plaque	No apparent softening, discoloration, hemorrahage, mass or other lesion. Junction between the cortex and white matter is well demarcated. Patchy periventricular demyelination bilaterally extending from the caudal frontal lobe to the rostral occipital lobe. Demyelinating plaque in the right proximal middle cerebellar peduncle. Normal straitum, lentiform nucleus, hippocampus and thalamus. No atrophy of the cerebellar folia. The basilar vasculature shows severe atherosclerosis.	Perivascular demyelination characterized by a variably decreased axonal loss and complete demyelination, severe gliosis and oligodendrocyte loss, no associated macrophage activity or perivascular lymphocytic cuffing, adjacent white matter and cortex are unremarkable.	Perivascular white matter	RRMS	Respiratory failure
3805	70	M	Frontal normal appearing white matter	No apparent softening, discoloration, hemorrahage, mass or other lesion. Mild atrophy of the frontal and temporal lobes. Junction between the cortex and white matter is well demarcated. Lateral cerebral ventricles are normal in shape and size. Normal striatum, lentiform nucleus, and thalamus. No atrophy of the cerebellar folia. No significant atherosclerosis.	Neocortex shows normal neuronal cellularity, no extracellular spongiosis, no evidence of neuritic or neurofibrillary pathology, hippocampus shows a very rare neurofibrillary tangle but is otherwise normal, no evidence of degeneration, hypoxic injury hemorrhage or inflammation	Hippocampus and temporal cortex	Acute renal failure, Type1 diabetes, Brain normal	Acute renal failure

3861	81	F	Frontal normal appearing white matter	No apparent, softening, discoloration, hemorrhage, mass or other lesion. No atrophy of the frontal and temporal lobes. Junction between the cortex and white matter is well demarcated. Lateral cerebral ventricles are normal in shape and size. Normal striatum, lentiform nucleus, and thalamus. No atrophy of the cerebellar folia. The basilar cerebral vasculature shows mild atherosclerosis.	Neocortex and hippocampus show normal neuronal cellularity, no extracellular spongiosis, no evidence of neurodegenerative disease, substantia nigra is unremarkable	Hippocampus, temporal cortex and substantia nigra	Chronic obstructive pulmonary disease, pneumonia, osteoporosis, tuberculosis, Brain normal	Chronic obstructive pulmonary disease, progressive weakness, pneumonia
3912	80	M	Frontal normal appearing white matter	No apparent, softening, discoloration, hemorrhage, mass or other lesion. No cerebral atrophy. Junction between the cortex and white matter is well demarcated. Lateral cerebral ventricles are normal in shape and size. Normal striatum, lentiform nucleus, and thalamus. No atrophy of the cerebellar folia. No significant atherosclerosis.	Hippocampus show few rare neurofibrillary tangles, no evidence of neuronal loss or neuritic plaque formation, temporal cortex is unremarkable, no evidence of metastatic carcinoma	Hippocampus and temporal cortex	Lung cancer, hypertension Brain normal.	Lung cancer, progressive respiratory insufficiency, respiratory failure
4064	75	F	Frontal normal appearing white matter	No evidence of metastatic cancer or any other softening, discoloration, hemorrhage, mass or other lesion. No cerebral atrophy. Junction between the cortex and white matter is well demarcated. Lateral cerebral ventricles are normal in shape and size. Normal striatum, lentiform nucleus, and thalamus. No atrophy of the cerebellar folia. No signs of atherosclerosis in the basilar cerebral vasculature.	Neocortex shows normal neuronal cellularity, no evidence of neurodegeneration, infarction, hypoxia or metastatic carcinoma, hippocampus is normal with no changes or inclusion in CA1 region	Hippocampus and temporal cortex	Cirrhosis, nonalcoholic steatohepatitis, hypothyroidism, depression, hypertension, renal failure acute anemia, Brain normal.	Laennec's cirrhosis, liver failure, progressive weakness
4135	57	M	Frontal normal appearing white matter	No apparent softening, discoloration, hemorrhage, mass or other lesion. No evidence of metastatic carcinoma and no grossly identifiable evidence of stroke. Mild atrophy of the frontal lobes. Junction between the cortex and white matter is well demarcated. Lateral cerebral ventricles are normal in shape and size. Normal striatum, lentiform nucleus, and thalamus. No atrophy of the cerebellar folia. No signs of atherosclerosis in the basilar cerebral vasculature.	Neocortex shows normal neuronal cellularity and normal subcortical white matter. Neocortex and hippocampus shows no evidence of neurodegeneration or hypoxia, no evidence of stroke or metastatic disease.	Hippocampus and temporal cortex	Hypertension, seizure disorder, acute hypoxia, chronic obstructive pulmonary disease, history of recent stroke (verbal only) Brain normal	-
