# Supplementary figures and images for: Inflammation induces neuro-lymphatic protein expression in multiple sclerosis brain neurovasculature
Source: J Neuroinflammation. 2013 Oct 14;10:125. doi: 10.1186/1742-2094-10-125 (PMC3854084; doi:10.1186/1742-2094-10-125)

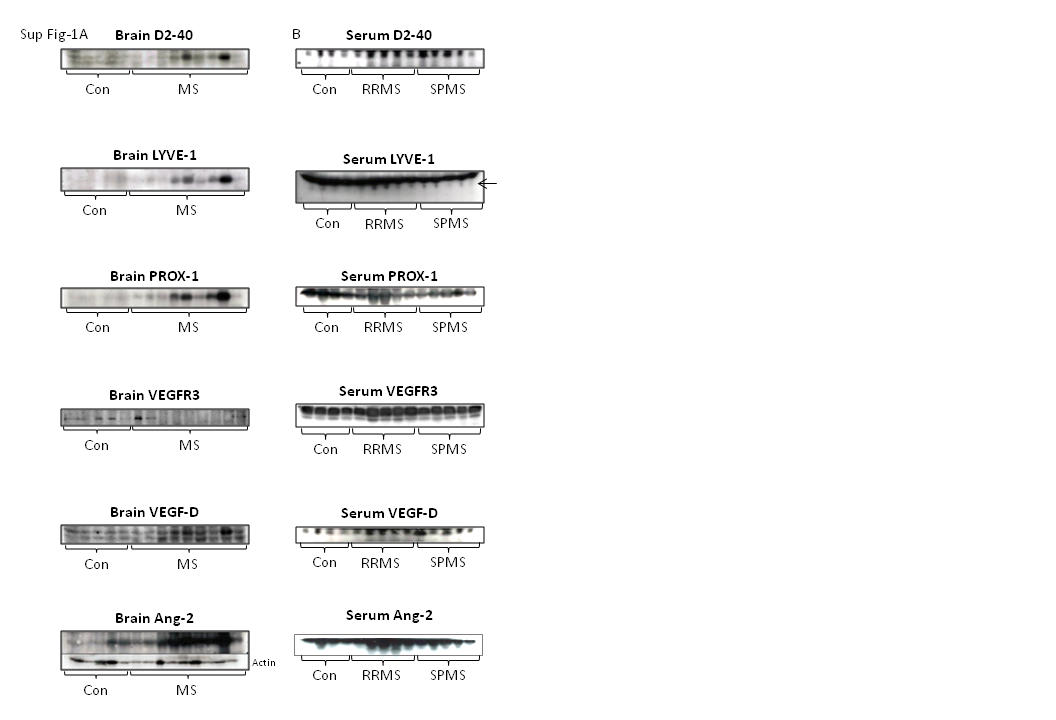

Supplement: Additional file 2: Figure S1 — (A) Western blot images of D2-40, LYVE-1, Prox-1, VEGFR-3, VEGF-D, and Ang-2 in control and RRMS brain tissue samples. Con n=5, RRMS n=9. (B) Western blot images of D2-40, LYVE-1, Prox-1, VEGFR-3, VEGF-D, and Ang-2 in control, RRMS, and SPMS serum samples. [file 1742-2094-10-125-S2.tiff]

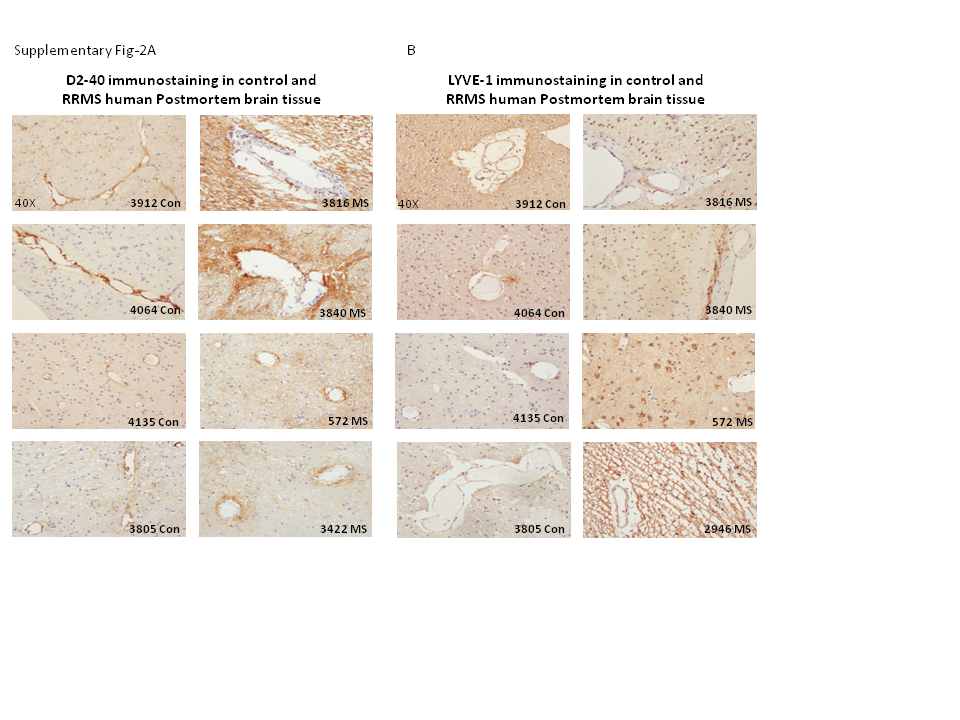

Supplement: Additional file 3: Figure S2 — (A) Additional D2-40 immunostained pictures of human postmortem control brain samples (3912, 4064, 4135, and 3805) and MS brain samples (3816, 3840, 572, and 3422). D2-40 immunostaining is intense and focused at the perivascular endothelial inflammatory region and parenchyma in all MS samples. (B) Additional LYVE-1 immunostained pictures of human postmortem control samples (3912, 4064, 4135, and 3805) and MS brain samples (3816, 3840, 572, and 2946). Brain parenchyma stained positive for intensively positive for LYVE-1 in MS sample 2946. Magnification 40×. [file 1742-2094-10-125-S3.tiff]

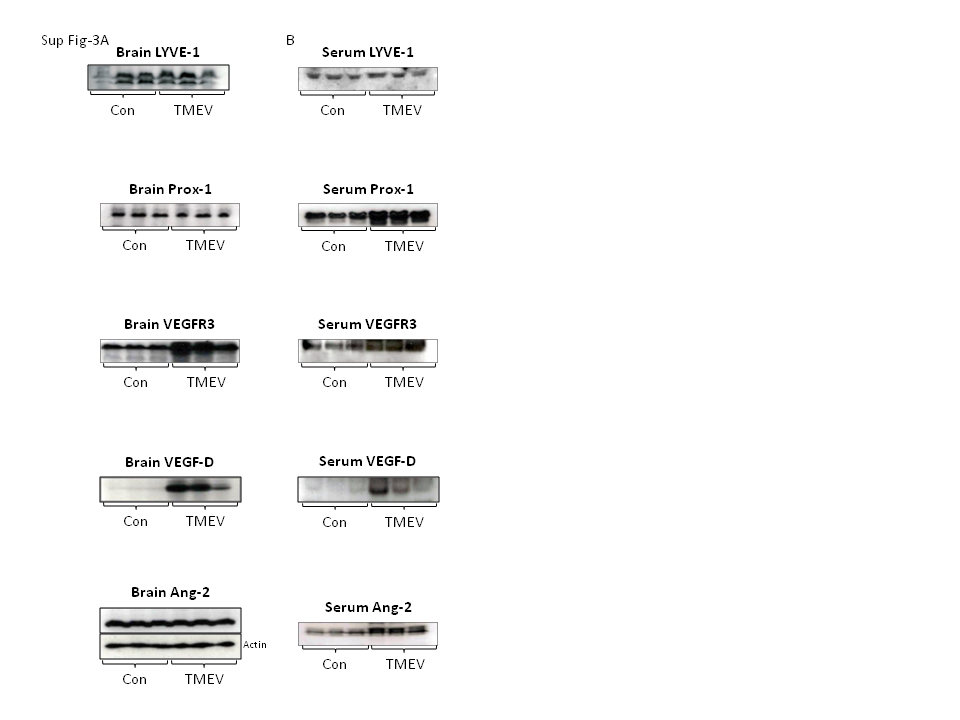

Supplement: Additional file 4: Figure S3 — (A) Western blot images of LYVE-1, Prox-1, VEGFR-3, VEGF-D, and Ang-2 in randomly chosen control and TMEV-IDD mice brain tissue samples. Con n=3, TMEV-IDD n=3. (B) Western blot images of LYVE-1, Prox-1, VEGFR-3, VEGF-D, and Ang-2 in corresponding serum samples from control and TMEV-IDD mice. [file 1742-2094-10-125-S4.tiff]

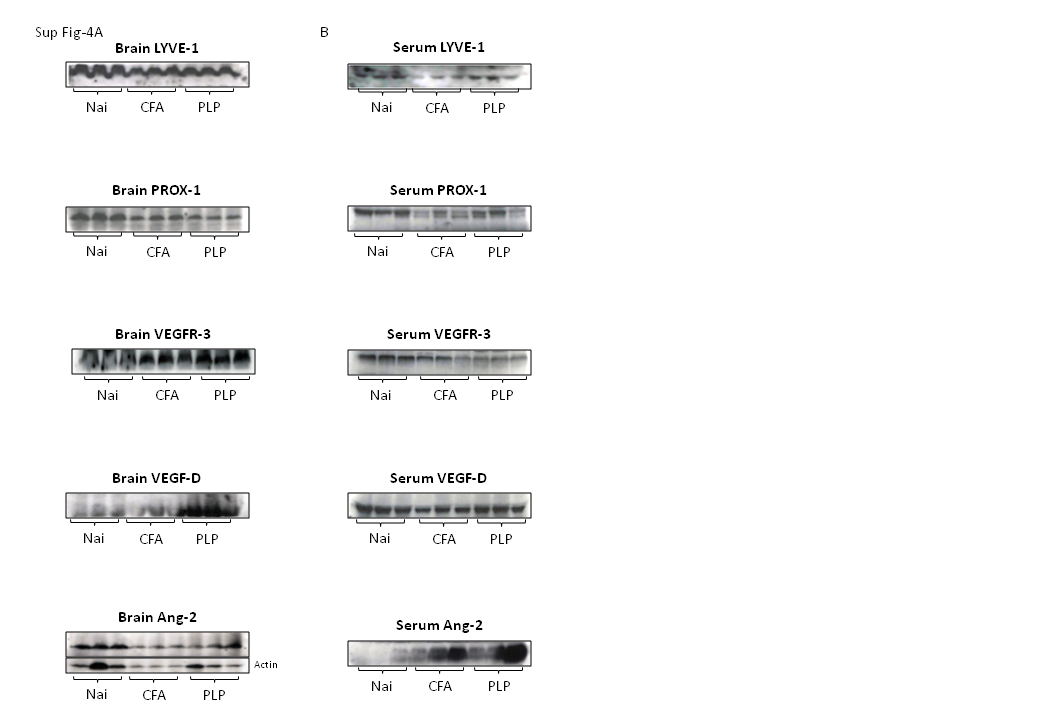

Supplement: Additional file 5: Figure S4 — (A)Western blot images of LYVE-1, Prox-1, VEGFR-3, VEGF-D, and Ang-2 in randomly chosen naïve, CFA-treated, and CFA+PLP-EAE mice brain tissue samples. Naïve n=3, CFA n=3, CFA+PLP-EAE n=4. (B) Western blot images of LYVE-1, Prox-1, VEGFR-3, VEGF-D, and Ang-2 in corresponding serum samples from naïve, CFA, and CFA+PLP-EAE mice. [file 1742-2094-10-125-S5.tiff]

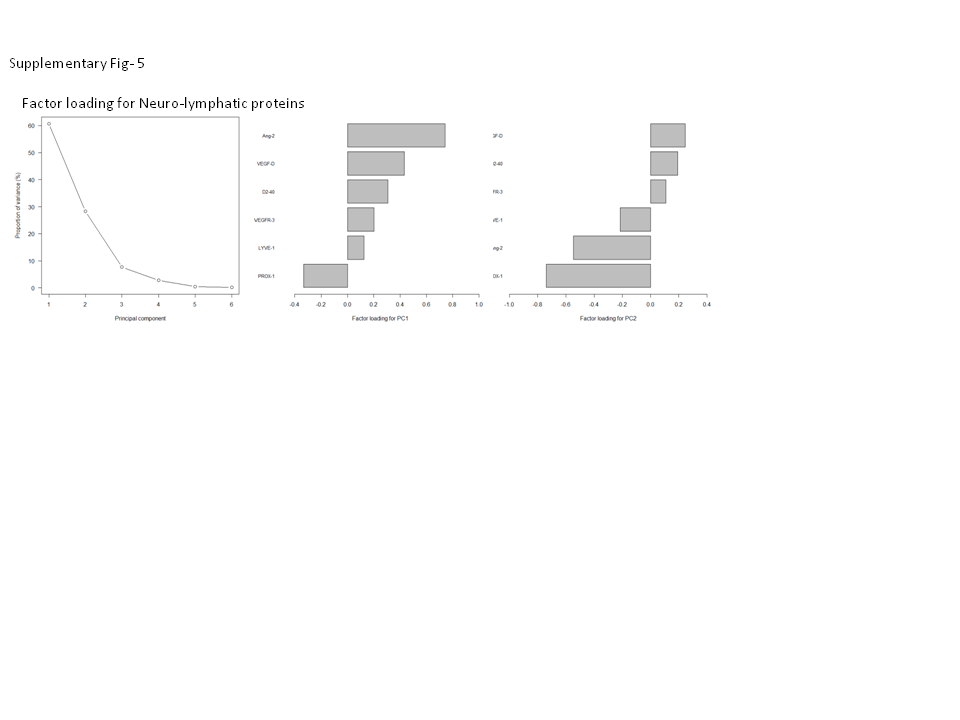

Supplement: Additional file 6: Figure S5 — Plot of the eigenvalues that reflect the variance of the principal components showed that >90% of the variance in this matrix of lymphatic proteins is contained in the first two principal components (PC1, 61%; PC2, 28%). The factor loading on the eigenvalues for PC1 and PC2 reflect the amount of variance shared by the parameter with the PC1 and PC2 values. [file 1742-2094-10-125-S6.tiff]

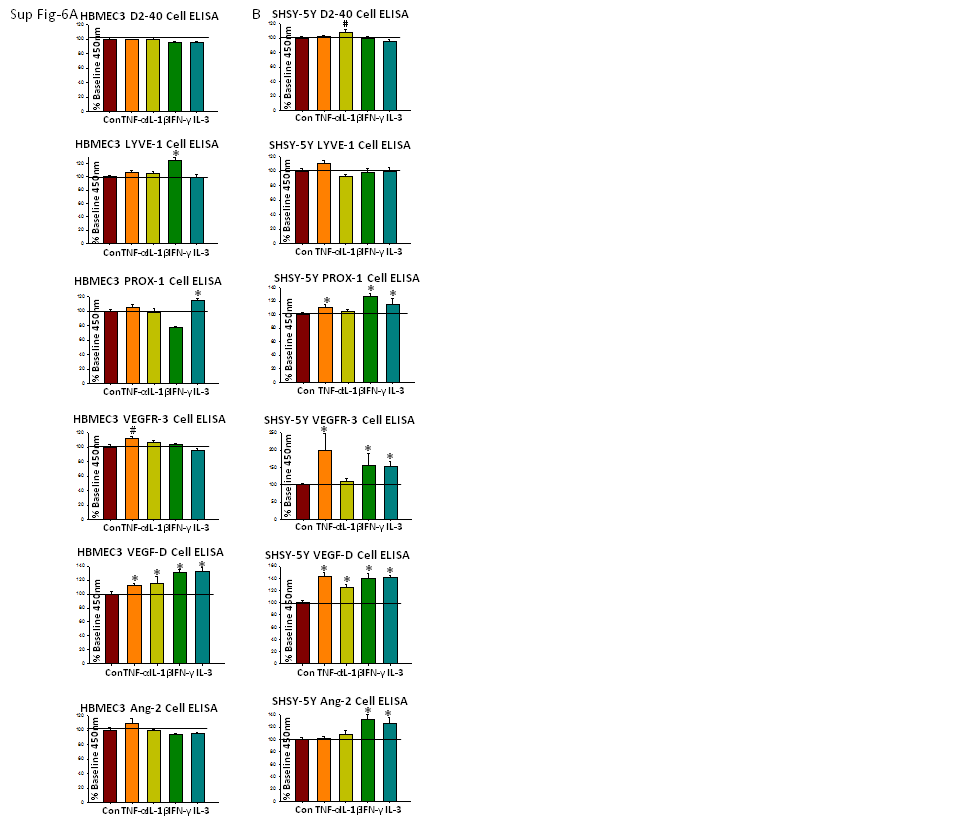

Supplement: Additional file 7: Figure S6 — (A) Bar graphs showing the cytokine (TNF-α, IL-1β, IFN-γ, and Il-3) mediated neuro-lymphatic protein expression profiles in HBEMC-3 brain endothelial cells. (B) Bar graphs showing the cytokine (TNF-α, IL-1β, IFN-γ, and Il-3) mediated neuro-lymphatic protein expression profiles in SYSY-5Y neurons. [file 1742-2094-10-125-S7.tiff]
